# The beam transport system for the Small Quantum Systems instrument at the European XFEL: optical layout and first commissioning results

**DOI:** 10.1107/S1600577522012085

**Published:** 2023-02-03

**Authors:** Tommaso Mazza, Thomas M. Baumann, Rebecca Boll, Alberto De Fanis, Patrik Grychtol, Markus Ilchen, Jacobo Montaño, Valerija Music, Yevheniy Ovcharenko, Nils Rennhack, Daniel E. Rivas, Aljoscha Rörig, Philipp Schmidt, Sergey Usenko, Pawel Ziołkowski, Daniele La Civita, Maurizio Vannoni, Harald Sinn, Barbara Keitel, Elke Plönjes, Ulf Fini Jastrow, Andrey Sorokin, Kai Tiedtke, Klaus Mann, Bernd Schäfer, Niels Breckwoldt, Sang-Kil Son, Michael Meyer

**Affiliations:** a European XFEL, Holzkoppel 4, 22869 Schenefeld, Germany; b Deutsches Elektronen-Synchrotron DESY, Notkestr. 85, 22607 Hamburg, Germany; c Department of Physics, University of Kassel, Heinrich-Plett-Straße 40, 34132 Kassel, Germany; d IFNANO Institut für Nanophotonik Göttingen e.V., Hans-Adolf-Krebs-Weg 1, 37077 Göttingen, Germany; e Center for Free-Electron Laser Science CFEL, Deutsches Elektronen-Synchrotron DESY, Notkestr. 85, 22607 Hamburg, Germany; f Department of Physics, Universität Hamburg, Notkestr. 9–11, 22607 Hamburg, Germany; g The Hamburg Centre for Ultrafast Imaging, Luruper Chaussee 149, 22761 Hamburg, Germany; RIKEN SPring-8 Center, Japan

**Keywords:** free-electron lasers, X-ray beam transport, microfocusing, atomic, molecular and optical science

## Abstract

The Small Quantum Systems instrument is one of the six operating instruments of the European XFEL, dedicated to the atomic, molecular and cluster physics communities. The instrument started its user operation at the end of 2018 after a commissioning phase. The design and characterization of the beam transport system are described here. The X-ray optical components of the beamline are detailed, and the beamline performances, transmission and focusing capabilities are reported. It is shown that the X-ray beam can be effectively focused as predicted by ray-tracing simulations. The impact of non-ideal X-ray source conditions on the focusing performances is discussed.

## Abstract 2

The soft X-ray beam from the SASE3 source at the European XFEL is delivered to the SQS instrument by a beam transport system. The system layout and performances are reported.

## 1. Introduction

The Small Quantum Systems (SQS) instrument at the European XFEL (Decking *et al.*, 2020 ▸) is dedicated to the investigation of atoms, molecules, clusters and biomolecules in the gas phase. It uses the beam from the SASE3 soft X-ray undulator between the carbon and argon *K* edges, in the range 260–3200 eV. The instrument, described in detail by Mazza *et al.* (2012 ▸) and Meyer *et al.* (2023 ▸), was commissioned in 2018 (Meyer *et al.*, 2023 ▸) and the results of the first successful user experiments have been published (Kastirke *et al.*, 2020*a* ▸,*b* ▸; Eichmann *et al.*, 2020 ▸; Mazza *et al.*, 2020 ▸; LaForge *et al.*, 2021 ▸; Jahnke *et al.*, 2021 ▸; Li *et al.*, 2021 ▸, 2022*a* ▸,*b* ▸; Boll *et al.*, 2022 ▸; Feinberg *et al.*, 2022 ▸). Experiments at the SQS instrument exploit the intense, short and coherent X-ray pulses for non-linear phenomena studies (Mazza *et al.*, 2020 ▸; Eichmann *et al.*, 2020 ▸; LaForge *et al.*, 2021 ▸; Boll *et al.*, 2022 ▸), for time-resolved experiments following dynamical processes on the femto­second timescale (Kastirke *et al.*, 2020*b* ▸; Jahnke *et al.*, 2021 ▸; Grychtol *et al.*, 2021 ▸; Rivas *et al.*, 2022 ▸) and for investigations using coherent scattering techniques (Ekeberg *et al.*, 2022 ▸; Feinberg *et al.*, 2022 ▸). This paper is dedicated to the description of the X-ray beam transport system in use during the commissioning phase and the first three user runs until the beginning of 2020, and reports on its performance, evaluated from the results obtained during its commissioning. It comprises three main sections. In Section 2, the optical layout of the beamline is shown and the different beam transport configurations are described. In Section 3, we report on the calculated and measured beamline transmission for the different beam transport configurations, which sorts them according to the different use cases. In Section 4, we report on the micro-focusing performances of the system, which have been both simulated by ray tracing and measured by direct as well as indirect methods.

## 2. Optical layout of the beamline

**Figure 1:**
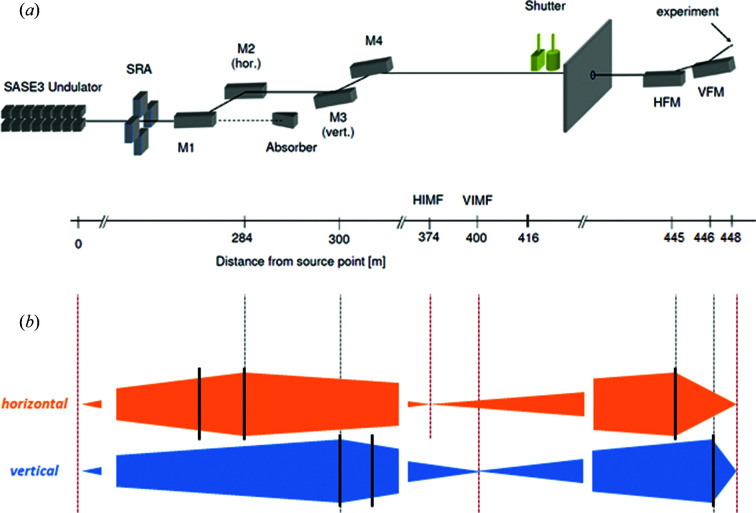
(*a*) Schematic representation of the beam transport layout of the SQS branch of the SASE3 beamline (Tschentscher *et al.*, 2017 ▸), which is described in detail in the text. The distance of each optical element to the source point is given at the bottom of the scheme. (*b*) The beam propagation conditions are schematically represented for both the vertical and the horizontal directions. The beam diverges from the source point until it reaches the first focusing element, which is M2 in the horizontal and M3 in the vertical direction. The beam converges horizontally and vertically on two different positions along the beamline, labeled HIMF and VIMF (horizontal and vertical intermediate focus, respectively), located at 90 m and 100 m distance from the respective mirrors. These positions are then the virtual sources imaged by the HFM (horizontal focusing mirror) and VFM (vertical focusing mirror) into the focal position where the experiment takes place. The dashed vertical lines indicate the position of the focusing mirrors and of the (virtual) sources and images in the vertical and horizontal directions.

The optical system transporting and focusing the free-electron laser (FEL) radiation extends over more than 400 m from the last SASE3 undulator section to the SQS experiment interaction region. The main FEL beam parameters are listed in Table 1 ▸. The optical system, schematically represented in Fig. 1 ▸(*a*), consists of six high-quality grazing-incidence mirrors, which are listed along with their characteristics in Table 2 ▸. The surface error of the mirrors, in the nm range, is at the state of the art, especially for such long substrates (Vannoni & Freijo-Martin, 2017 ▸, 2019 ▸). The first four of these mirrors, named M1, M2, M3 and M4, are located in an underground tunnel. The last two, named horizontal focusing mirror (HFM) and vertical focusing mirror (VFM), build up a Kirkpatrick–Baez (KB) mirror system and are located in the SQS instrument hutch. All mirrors are coated with a 50 nm-thick layer of boron carbide (B_4_C).

The mirrors M1 and M2 constitute a horizontal chicane, which filters out the high-photon-energy background (spontaneous radiation, *bremsstrahlung* radiation) as well as contributions from higher harmonics of the FEL fundamental wavelength. The grazing-incidence angle of both mirrors can be set to any value between 9 and 20 mrad by changing their pitch and their relative transverse position. Working points have been defined at 9, 13 and 20 mrad. The beam divergence increases monotonically with the wavelength (Table 1 ▸), so that at low photon energies the geometric transmission is effectively increased by increasing the incidence angles, which give a larger projected clear aperture. At the same time, the range of the transmitted photon energy by the reflectivity of the mirrors increases with decreasing incidence angle, with a cutoff changing from about 1.4 keV to >3 keV for an incidence angle varying from 20 mrad to 9 mrad. M1 is a flat mirror; M2 is equipped with a mechanical bender inducing a cylindrical curvature (Vannoni *et al.*, 2016 ▸) which is varied with the incidence angle to focus the beam horizontally at a distance of 90 m from the mirror, or 374 m from the source. This feature makes the size of the beam footprint at the position of HFM comparable with the projected clear aperture of the mirror itself (see Fig. 1 ▸ and Table 2 ▸).

The mirrors M3 and M4 constitute a vertical chicane. Two different substrates can be placed in the M3 position depending on the beam wavelength, with an incidence angle of 20 mrad for low photon energies (low-energy pre-mirror, LEPM) and 9 mrad for high photon energies (high-energy pre-mirror, HEPM). Both pre-mirrors have a spherical shape with a fixed radius, to focus the beam at a distance of 100 m from the mirror. M4 is a plane mirror which can be replaced by a variable line spacing (VLS) grating if a monochromatic beam is required by the beam users; the spectral band is selected in this case by a vertical slit located at the focal position of M3. The grating and the slit constitute the SASE3 soft X-ray monochromator (Gerasimova *et al.*, 2022 ▸). In addition, the focusing provided by M3 narrows the beam also vertically, such that, similarly to the horizontal direction, the beam size at the position of VFM is made compatible with the projected clear aperture of the mirror itself (see Fig. 1 ▸ and Table 2 ▸).

Depending on the experimental requirements in terms of photon energy range, various beam transport configurations are then defined, combining the different set points of the horizontal and vertical chicane. The beam transport performances regarding transmission are presented for selected configurations in Section 3. All the transport configurations deliver the beam to the same position in the instrument hutch, where it is focused into the experimental region by the last two mirrors.

The HFM and VFM mirrors are a pair of elliptical substrates with fixed radii, installed in a KB configuration. They are dedicated to microfocusing the beam into the experimental region of the SQS instrument. Their design parameters are *a*_HFM_ = 28 m, *b*_HFM_ = 9.41 m, *a*_VFM_ = 15 m, *b*_VFM_ = 5.34 m, where *a* and *b* are, respectively, the semi-major and semi-minor axes of the elliptical profiles in the meridional direction. These correspond to a source distance to the mirror center *p*_0_ = 52.7 m (28 m) and an image distance *q*_0_ = 3.3 m (2.0 m) for the HFM (VFM) with an incidence angle at the mirror center of θ_0_ = 9 mrad for both HFM and VFM (Peatman, 1997 ▸). The mirrors are used to image the virtual sources given by the horizontal and vertical foci generated along the tunnel by M2 and M3, called intermediate foci (IMFH, IMFV), into the experimental region of the SQS instrument. The mismatch between the respective *p* parameters, stated above, and the distances IMFH − HFM = *p*_H_ = 72 m and IMFV − VFM = *p*_V_ = 46 m is compensated, approximating the elliptical profile to a cylindrical profile, by detuning the incidence angle from the set value θ_0_ by Δθ, with (Peatman, 1997 ▸) 

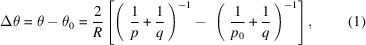

resulting in the incidence angles listed in Table 2 ▸. Because of this adjustment, the beam profile is affected by minor spherical aberrations. The impact of these on the focal spot size is discussed in Section 4.

The KB mirrors with fixed radii have been recently replaced in the SQS instrument by a pair of mechanically bendable mirrors, aiming for better focusing performances and higher operational flexibility. The new KB system will be described elsewhere (Mazza *et al.*, 2023 ▸).

## 3. Beamline transmission

The beam transport layout described in Section 2 is designed to provide high transmission for the X-ray beam for a photon energy range spanning more than one order of magnitude (Table 1 ▸). The transmission is limited on the low-photon-energy side by the large beam size, due to the increased divergence, exceeding the finite size of the mirrors, and on the high-energy side by the cutoff determined by the grazing-incidence angle.

The transmission was calculated for different beam transport configurations as the product of the combined reflectivity of the six mirrors and the geometric acceptance of the beamline. The mirror reflectivity was calculated using the X-ray Database of the Center for X-ray Optics (The Center for X-ray Optics, 2022 ▸). Photon beams with horizontal polarization and energies between 0.25 and 4 keV were reflected by a single layer of B_4_C with a thickness of 50 nm on a Si substrate under incidence angles of 9, 13 and 20 mrad. The density of the substrate is assumed to be equal to the bulk value. For the coating density, a value of 2.37 g cm^−3^ was used. The coating density of the mirrors was measured by X-ray reflectometry using a laboratory source with a sub-nm accuracy on smaller superpolished silicon substrates coated together with each mirror (Störmer *et al.*, 2018 ▸). The geometric acceptance of the beamline was obtained by analytically calculating the propagation of a beam with source size and divergence as given in Table 1 ▸ through the optical layout schematically represented in Fig. 1 ▸.

The transmission was also experimentally determined by simultaneously measuring the energy per pulse upstream and downstream of the six mirrors by means of gas monitor detectors (XGMs) (Baumann *et al.*, 2023 ▸; Sorokin *et al.*, 2019 ▸), one located before any optical element and the other in the experimental hutch after the last reflecting mirror. The measurements were performed for selected beam transport configurations over a wide range of photon energies.

**Figure 2:**
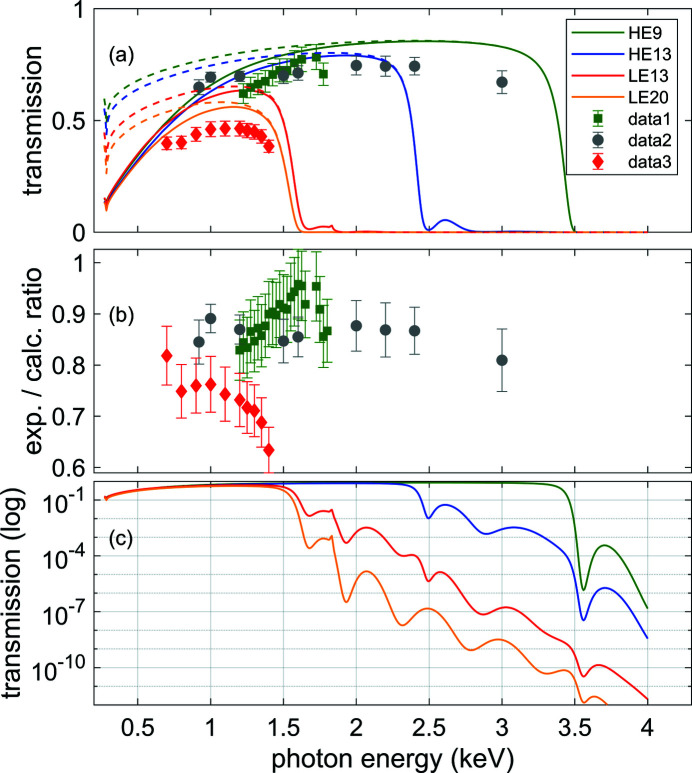
(*a*) Calculated and measured beamline transmission, including all six optical elements, as a function of photon energy for different beam transport configurations. The dashed curves represent the reflectivity contribution to the transmission, whereas the full lines represent the total transmission given by the product of the reflectivity and the geometric acceptance contributions. Different colors of the curves correspond to different incidence angle settings for the horizontal (H) and vertical (V) chicanes. Yellow: *H* = 20 mrad, *V* = 20 mrad; red: *H* = 13 mrad, *V* = 20 mrad; blue: *H* = 13 mrad, *V* = 9 mrad; green: *H* = 9 mrad, *V* = 9 mrad. The points represent transmission measurements. Green squares (‘data1’): *H* = 9 mrad, *V* = 9 mrad; gray circles (‘data2’): *H* = 9 mrad, *V* = 9 mrad, probing reflectivity only (see text); red diamonds (‘data3’): *H* = 13 mrad, *V* = 20 mrad. (*b*) Ratio between the measured and the respective calculated transmission values shown in (*a*). (*c*) Calculated beamline transmission, in logarithmic scale, showing the transmission above the cut-off energy, which determines the fraction of the original higher harmonics contribution from the undulator, which is delivered to the experiment (Baumann *et al.*, 2023 ▸).

Measured and calculated transmission values are shown in Fig. 2 ▸(*a*). The transmission is high over a large range, and the comparison shows a good agreement between predicted and observed beamline transmission over the whole range.

The curves represent the calculated beamline transmission for the four standard beam transport configurations, in which either the HEPM or the LEPM is used in the vertical chicane; in combination with the HEPM, the horizontal chicane is set to an incidence angle of either 9 or 13 mrad, whereas in combination with the LEPM the horizontal chicane is set to an incidence angle of either 13 or 20 mrad.

The full lines represent the transmission given by the product of the reflectivity and geometric acceptance contributions. The dashed lines give the reflectivity contribution to the overall transmission, showing that for photon energies above 2 keV, where the beam size is small enough, the geometric acceptance is essentially equal to one. The geometric acceptance at low photon energies drops because of the increasing beam size given by its larger divergence (Table 1 ▸). At high energies, the transmission is cut by a drop in reflectivity above a photon energy value which depends on the incidence angle.

The points represent measurements performed under different beam transport configurations, to be compared with the corresponding calculated curves. The measurements have been performed changing also the opening conditions of a double slit system located right after the source, before the upstream XGM, called the synchrotron radiation aperture (SRA). With a small enough SRA, only the central part of the beam is propagated, so that geometric losses play no role in determining the measured transmission. The measurement performed under such conditions, with an SRA of 1 mm × 1 mm, is labeled ‘data2’ and can then be compared with the calculated reflectivity contribution. The ratio between the measurements and the calculated transmission is shown in Fig. 2 ▸(*b*). The measured transmission is on average between 10 and 20% lower than calculated, likely due to degradation of the mirror coatings induced by X-ray exposure. The stronger reduction of the transmission observed when using the LEPM would be in this case consistent with the more intensive use that this substrate had undergone at the time of the measurements. Also, the reflectivity of the peripheral parts of the mirrors, which are not probed by the measurement made using a narrow SRA, is possibly less degraded due to the milder X-ray exposure compared with the central part, which could explain the higher ratio between experiment and calculations observed for the measurement with larger SRA (‘data1’) compared with the measurement with narrower SRA (‘data2’).

Fig. 2 ▸(*c*) shows the calculated transmission curves in a logarithmic scale. This representation gives a semi-quantitative picture of how the different beam transport configurations are effective in suppressing contributions from higher harmonics of the FEL fundamental. An experimental investigation quantifying these contributions from the SASE3 undulator will be published elsewhere (Baumann *et al.*, 2023 ▸).

## 4. Focusing performance

The expected spatial distribution of the X-rays in the focal spot produced by the KB mirrors has been calculated by ray-tracing simulations using the *SHADOW* code (Sanchez del Rio *et al.*, 2011 ▸). The source was modeled as a Gaussian beam originating at the end of the last undulator cell. A source with a finite longitudinal extension, accounting for the situation in which the FEL exponential power growth saturates before the last undulator cell is reached, was modeled as the superposition of point-like sources located at the end of each of the last *n* undulator cells, with the number *n* as well as the relative intensity of the sources set as variable parameters in the simulations. The source size and divergence were set based on the expressions given in Table 1 ▸.

The beam was propagated along the SASE3 beamline, described in Section 2 (see Fig. 1 ▸ and Table 2 ▸), adjusting the incidence angle of the HFM and VFM mirrors to compensate the mismatch between their design profile and the beamline geometry. The contribution from the measured polishing error of the mirrors (see Table 2 ▸) was explicitly included in the simulations as an additional surface error. As ray-tracing simulations do not include diffraction effects, to account for the diffraction contribution given by the finite size of the mirrors to the beam profile at the focal position a random distribution was added to the rays’ position in both directions of the focal plane. The probability for this distribution follows the single slit diffraction intensity and is given by 

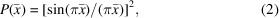

with 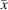 = CAθ/(λ *q*)*x* where *q* is the mirror–focus distance, λ is the X-ray wavelength, CA is the mirror clear aperture, θ is the grazing-incidence angle and *x* is the displacement from the beam axis in either the horizontal or the vertical direction.

**Figure 3:**
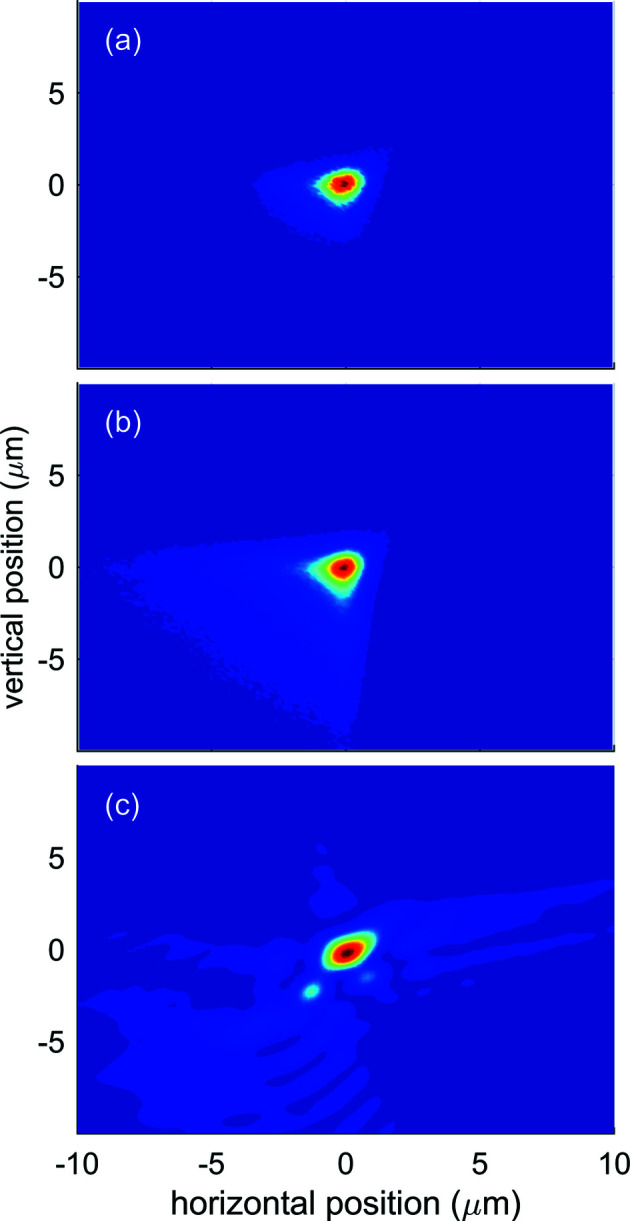
Ray-tracing simulations results compared with a wavefront measurement of the beam profile in the focal position at *h*ν = 1050 eV. For all three beam profile images the color scale goes from 0 (blue) to 1 (red), as each image is normalized to the maximum value. (*a*) Beam profile from ray-tracing simulations performed assuming a Gaussian-shaped source with size and divergence as per Table 1 ▸ with no extension in the longitudinal direction. (*b*) Beam profile from ray-tracing simulations performed assuming a source extending over the last ten undulator cells (see text). (*c*) Beam profile as obtained from the wavefront sensor measurement.

**Figure 4:**
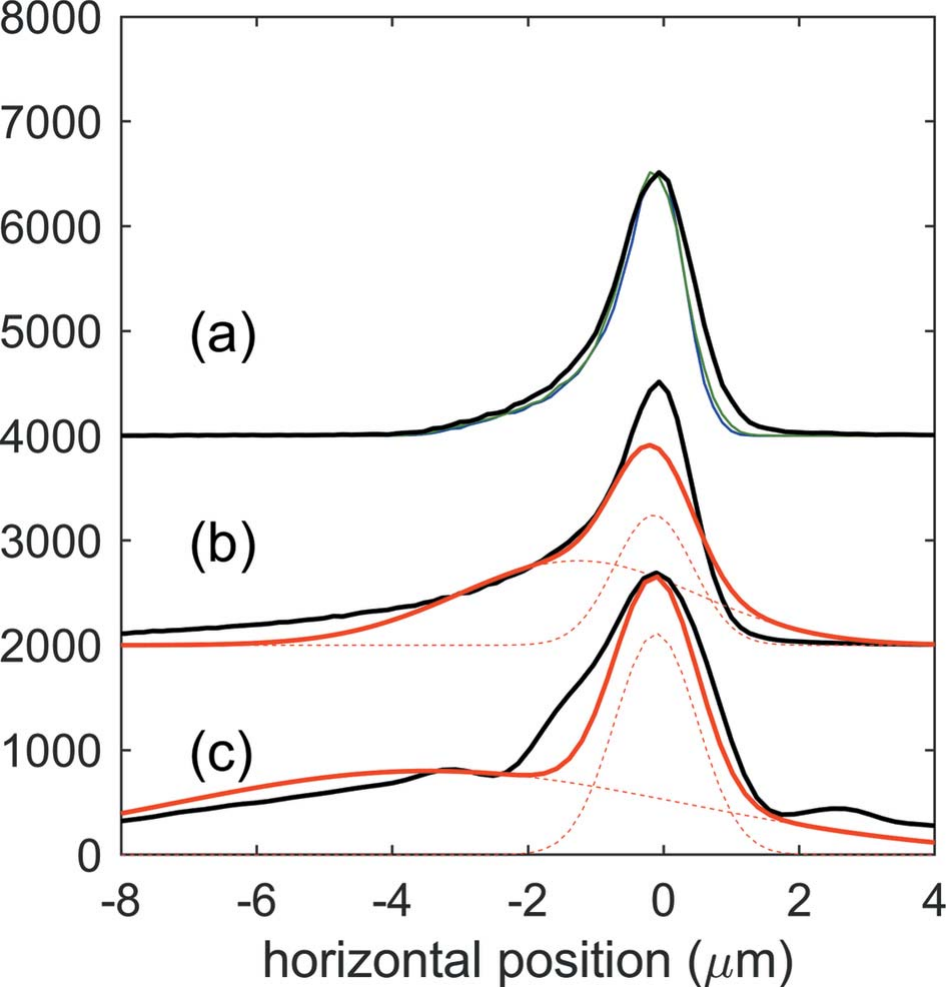
Curves (*a*), (*b*) and (*c*) in black are the projection in the vertical direction of the focal beam profiles shown in Figs. 3 ▸(*a*), 3(*b*) and 3(*c*), respectively. The projection of the profile obtained from a simulation assuming a source without extension in the longitudinal direction [(*a*), black] is compared with the projection of the same profile, assuming no diffraction effects from the finite length of the mirrors [(*a*), green], and assuming no surface error and no diffraction effects [(*a*), blue]. The projection of the profiles obtained from a simulation assuming an extended source over the last ten undulator cells [(*b*), black] and the projection of the profile measured by the wavefront sensor [(*c*), black] are compared with the projection of a fitting distribution made of the sum of two 2D Gaussian profiles [(*b*) and (*c*), red]. The projections of the individual Gaussian contributions are also plotted as dashed red lines.

The beam intensity profile in the focal position, simulated under the assumption of a source with no longitudinal extension located at the end of the last undulator cell for a photon energy of 1.05 keV, is shown in Fig. 3 ▸(*a*). The predicted beam size is approximately 1.5 µm full width at half-maximum (FWHM), matching the requirement for a micrometric focus which is necessary for performing non-linear studies. The polishing error and the finite mirror size diffraction contributions, estimated from the squared difference between the simulations with and without inclusion of the respective effects, amount to 0.55 and 0.73 µm FWHM, respectively (see Fig. 4 ▸). According to expression (2), diffraction effects dominate the beam size in the low end of the photon energy range and become smaller as the wavelength decreases. The asymmetric tail observed on the negative side in both horizontal and vertical directions is due to aberrations given by the deviation of the mirror surface profile from the ideal one, given by the mismatch between the elliptical shape of the HFM and VFM mirrors and the beamline geometry. This mismatch is compensated only in the first order by detuning the incidence angle of the focusing mirrors (see Section 2).

The FEL radiation generated by the undulators is commonly assumed to originate from the last cell of the undulator array, which in the case of SASE3 consists of 23 cells spaced 6.1 m from each other (Fig. 1 ▸). This point, identified as the radiation source, can be effectively focused in the experiment by an optical system based on reflective elements like the one described here, by adapting the radius in the case of bendable mirrors or by adapting the grazing-incidence angle as for the present case.

If the upstream cells yield non-negligible radiation, these contributions will be focused at different positions along the propagation direction, being delivered out of focus to the desired image position. Such a situation is realized when the FEL gain saturation level is reached at an undulator cell *n*_sat_ located upstream of the last undulator cell *N*, *n*_sat_ < *N*: the undulator gain curve *I*(*n*) grows less than exponentially for the downstream radiators, *I*(*n*)/*I*(*n* − 1) < *I*(*n* − 1)/*I*(*n* − 2) for *n* > *n*_sat_, and the radiation yield from each of the downstream cells becomes comparable, *I*(*n*) − *I*(*n* − 1) ≃ *I*(*n* − 1) − *I*(*n* − 2) for *n* > *n*_sat_ (Milton *et al.*, 2001 ▸).

Fig. 3 ▸(*b*) shows the beam intensity profile in the focal position simulating an extended source consisting of ten points located at the end of each of the last ten undulator cells, each radiating with the same intensity. This provides a rough modeling for the situation in which the FEL gain saturation is reached by the 14th of the 23 cells of the SASE3 undulator. Coherence effects are neglected in the ray-tracing simulations. The beam FWHM is approximately the same as in Fig. 3 ▸(*a*), but the asymmetric tail is much more pronounced, resulting in a broad, low-intensity underlying contribution with a size of several micrometres. The photon density distribution can be modeled as the sum of two contributions, a narrower one *G*1, with total integral *E*_*G*1_, width *w*_*G*1_ and maximum *f*_*G*1_, coming mostly from the imaging of the in-focus radiator, and a broader background *G*2 with total integral *E*_*G*2_, width *w*_*G*2_ and maximum *f*_*G*2_ yielded by the radiating cells located further away. Fitting the distribution with the superposition of two Gaussians (Fig. 4 ▸) shows that for the present simulation conditions the contribution from *G*2 is higher than that from *G*1 (*E*_*G*2_/*E*_*G*1_ = 1.89) but the contribution from *G*2 to the peak fluence is minor (*f*_*G*2_/*f*_*G*1_ = 0.21), because of the larger width of the second Gaussian with respect to the first (*w*_*G*2_ = 4.1 µm, *w*_*G*2_/*w*_*G*1_ = 3.0).

The experimental focusing conditions were optimized during the commissioning phase of the instrument. After aligning the beamline elements in the tunnel, focusing was optimized by fine tuning the incidence angle (pitch) of the fixed-radii KB mirrors and in addition by adjusting the roll of the HFM to get the two mirrors to be orthogonal to each other. For the horizontal (vertical) direction, around the nominal focal distance a variation of 1 µrad in the incidence angle would move the focus longitudinally by 0.38 mm (0.29 mm), and accordingly in the transverse direction by 6.6 µm (4 µm), so when adjusting the focal distance by several centimetres a translation in the order of 1 mm of the focal position in both directions in the beam plane would need to be accounted for. Ray-tracing simulations show that the aberrations given by the mismatch between the mirror profiles and the beamline geometry are minimized, albeit not completely removed, when the image distance set by the incidence angle detuning is equal to the nominal (design) image distance of the mirrors, which was set as the design distance for the position of the interaction region.

The optimization and then the characterization of the beam focusing was aided by different diagnostics tools.

### 4.1. Wavefront sensor

The spatial distribution of the beam intensity in the focal position was reconstructed from wavefront measurements performed using a Hartmann-type wavefront sensor (WFS) (Keitel *et al.*, 2016 ▸). The WFS is based on an array of 75 µm-diameter holes, electroformed on a quadratic pattern with a 250 µm pitch in a 20 µm-thick Ni foil, the Hartmann plate. The Hartmann plate is exposed to the beam 2.3 m downstream of the focal position and is placed upstream of the CCD chip coated with an EUV-to-VIS quantum converter P43 (1392 × 1040 pixels with 6.45 µm × 6.45 µm pixel size; field of view of 8.9 mm × 6.7 mm) at a distance of ∼200 mm.

As described in detail by Keitel *et al.* (2016 ▸), the plate splits the incoming beam into an array of sub-beams which are recorded by the CCD. The displacement of a spot centroid position with respect to a previously measured reference plane wave, divided by the camera–plate distance, yields the local radiation angle and thereby the local wavefront gradient inside one sub-aperture. The wavefront is reconstructed from these local gradients using mathematical algorithms. The extracted information allows one to compute relevant beam parameters as well as to back-propagate the beam spatial profile to the focal position.

Measurements were performed at a photon energy of *h*ν = 1.05 keV by attenuating the X-ray beam, optimized for high pulse energy, by several orders of magnitude, to preserve the Hartmann plate and the coated CCD camera from damage. A representative reconstructed single-shot intensity distribution of the X-ray beam in the focal plane is shown in Fig. 3 ▸(*c*). The photon beam distribution at the focus position was determined by numerically back-propagating the beam obtained from the WFS measurement of the wavefront phase and intensity after optimization of the focusing conditions. The optimization was performed by tuning the pitch and roll of the KB mirrors, aiming for minimizing the Zernike coefficients provided by the WFS algorithm which quantifies the optical aberrations of the wavefront. The intensity distribution is characterized by a sharp ‘hot-spot’ contribution *G*1, with a FWHM of 1.4 µm, superposed on a broad background *G*2 (FWHM = 9 µm), as given by the double 2D Gaussian fit (see Fig. 4 ▸).

We then describe the X-ray density distribution measured by the WFS using the same double Gaussian model described above; from a double Gaussian fit analysis performed on a data set of 20 single FEL shots we get the statistically more robust estimates for the parameters *w*_*G*1_ = 1.9 µm, *w*_*G*2_ = 8 µm, *w*_*r*_ = *w*_*G*2_/*w*_*G*1_ = 4.2, *E*_*G*2_/*E*_*G*1_ = 2.6, *f*_*r*_ = *f*_*G*2_/*f*_*G*1_ = 0.15.

### 4.2. Charge-state distribution based on light atoms

The size of the beam profile was also indirectly characterized by determining the incident photon fluence distribution from a quantitative comparison between theoretical and experimental charge-state distributions (CSDs) that are generated by X-ray multiphoton multiple ionization of Ar atoms. The CSDs, measured by ion time-of-flight mass spectrometry (Meyer *et al.*, 2023 ▸), are modeled using the *XATOM* package (Son *et al.*, 2018 ▸), which calculates the atomic electronic structure and simulates their ionization dynamics under intense FEL pulses, with calibrated beam parameters. We employed an extended version of *XCALIB* (Toyota *et al.*, 2019 ▸) to calibrate the fluence distribution at the focal spot, utilizing a range of pulse energies (Breckwoldt *et al.*, 2023 ▸). The numerical method is described in detail by Toyota *et al.* (2019 ▸). In brief, for a given photon energy the *XATOM* toolkit is used to calculate the ion yield for different charge states as a function of the photon fluence over a broad range. Instead of mapping the spatial fluence distribution as a function of position, *XCALIB* combines the theoretical CSDs to model the experimental CSDs with a few fitting parameters assuming a specific form of the spatial fluence profile in the focus position, giving access to the beam spatial distribution quantities, including the beam size.

We measured the CSDs of Ar atoms undergoing X-ray multiphoton multiple ionization at photon energies between 1200 and 1700 eV. For each photon energy, the pulse energy on target was tuned over a range of more than one order of magnitude between 0.1 and 2 mJ with the aid of a gas attenuator device (Sinn *et al.*, 2012 ▸). The theoretical CSDs are fitted to experimental CSDs with two different fluence distribution models, assuming that the beam spatial profile did not change when attenuating the X-ray beam, so retrieving a unique set of parameters for each photon energy and a relation between the experimental pulse energies delivered to the sample {*E*_pulse_} and the peak fluence in the focal plane {*F*_peak_}. The simplest model would be the beam spatial distribution described by a single Gaussian, so that the *E*_pulse_ values would be scaled to the *F*_peak_ values according to *F*_peak_ = 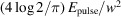 . The parameter *w*, representing the beam FWHM at the focal position, would be the only fitting parameter in this case. Based on the results from ray-tracing simulations and on the observation from the WFS measurement, following the method depicted by Toyota *et al.* (2019 ▸) we instead describe the fluence spatial distribution in the focal plane by a superposition of two Gaussian distributions with partial yields *E*_*G*1_ and *E*_*G*2_, with 

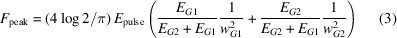

which, expressed by the terms introduced in the previous subsection, *f*_*r*_ = *f*_*G*2_/*f*_*G*1_, *w*_*r*_ = *w*_*G*2_/*w*_*G*1_ and *E*_*G*2_/*E*_*G*1_ = 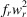 , reads 

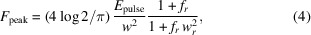

which is equivalent to equation (17*d*) of Toyota *et al.* (2019 ▸), as *E*_pulse_ is the pulse energy on target, so the quantity *T* entering the expression of Toyota *et al.* (2019 ▸) is set equal to 1. The scaling between the *E*_pulse_ values and the *F*_peak_ values in the focal plane is then given by three independent parameters: the width of the *G*1 Gaussian contribution *w* (= *w*_*G*1_), the ratio between the peak fluence of the two Gaussian contributions *f*_*r*_ and the ratio between the width of the two contributions *w*_*r*_. The photon budget is distributed between the two contributions according to *E*_*G*2_/*E*_*G*1_ = 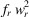 . The fluence distribution in the interaction region, which is limited along the beam direction *z* by the acceptance of the ion spectrometer, actually depends on the position *z* according to *w* = *w*_0_ [1 + (2*z*/*d*)^2^]^1/2^, where *d* is the depth of the beam focus. A comparison (not shown here) between the calculations based on a 3D modeling including the variation of the intensity distribution along the beam path and a simplified 2D calculation demonstrates that this variation can be safely neglected for the present case, and a model assuming constant conditions along the propagation axis can be used.

**Figure 5:**
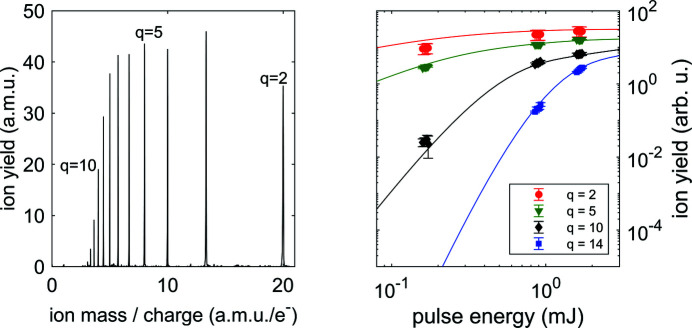
Left: a representative time-of-flight mass spectrum of multiphoton ionized Ar atoms. Photon energy was *h*ν = 1200 eV, pulse energy was *E*_p_ = 1.0 mJ, corresponding to 5 × 10^12^ photons per pulse. Right: measured CSDs as a function of pulse energy at *h*ν = 1200 eV for selected charge states compared with the best-fitting curves from the *XATOM* calculations, yielding the parameters reported in the first row of Table 3 ▸.

Fig. 5 ▸ shows an example time-of-flight mass spectrum from Ar at a given pulse energy and the measured CSDs of Ar for selected charge states over a range of pulse energies, at a photon energy of 1200 eV. The CSDs have been fitted by theory to yield the photon energy dependent parameters *w*, *f*_*r*_ and *w*_*r*_ reported in Table 3 ▸. The fitting procedure is described in detail by Breckwoldt *et al.* (2023 ▸).

### 4.3. Focusing performance results overview

The charge-state distribution characterization results based on multiphoton ionization are in very good quantitative agreement with both the outcome of the ray-tracing simulations and the results from the wavefront sensor characterization. Both experiments and simulations clearly show that the beam transport system can deliver the X-rays from the SASE3 undulator into the experiment focused down to below 1.5 µm FWHM, enabling one to perform non-linear studies on small quantum systems. With a pulse energy of 1 mJ and the parameters reported in Sections 4.1 and 4.2, an energy fluence of 0.12 to 0.16 mJ µm^−2^ is obtained in the focal position.

**Figure 6:**
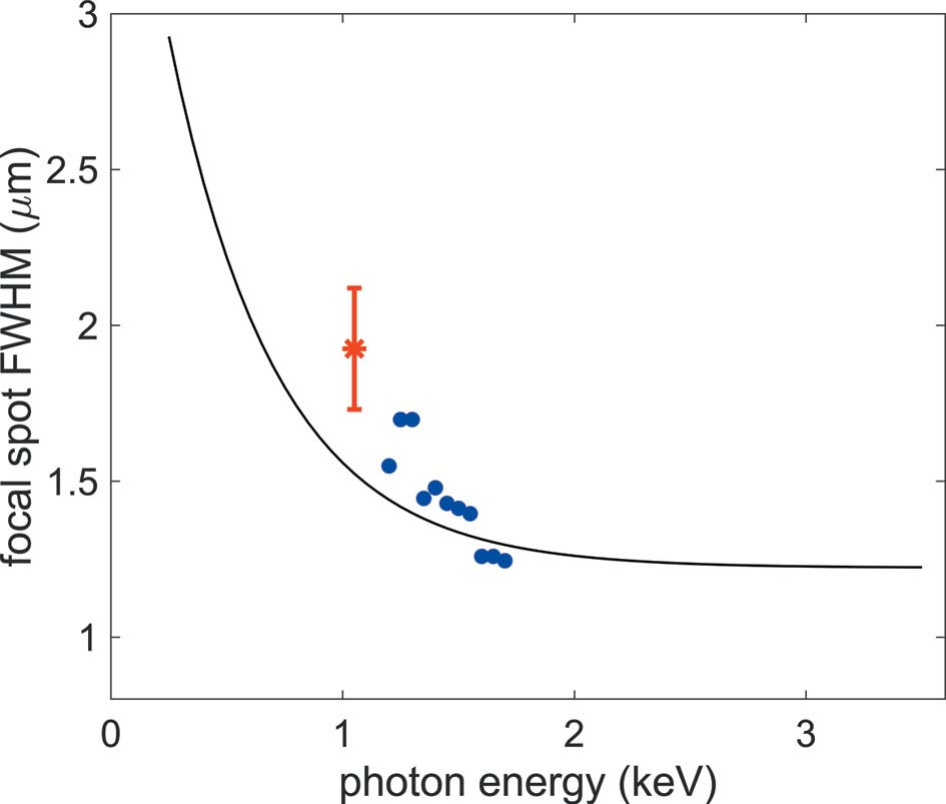
Black line: beam size (FWHM) as a function of photon energy estimated from the ray-tracing simulated intensity distribution under the assumption that the FEL source is located at the end of the last undulator, with no longitudinal extension. The simulations are performed accounting for the surface error of the mirrors and taking into account the diffraction effects, as described in the text. Red star: beam size from the WFS characterization, taken from the FWHM of the smaller Gaussian contribution to the double Gaussian fit of the 2D intensity distribution [see Figs. 3 ▸(*c*) and 4 ▸(*c*)]. Blue dots: beam size from the CSD characterization, taken from the FWHM of the smaller Gaussian contribution to the double Gaussian model for the fluence distribution used to fit the CSDs collected as a function of photon energy, see Table 3 ▸.

Using a double Gaussian fluence distribution for modeling the CSD of Ar ions and the WFS measurement, we get the indication that the beam profile is characterized by the presence of a narrow contribution with a linear size between 1 and 1.5 µm over a broader term with a size from two to three times larger. From the ray-tracing simulation results, it is natural to consider the narrow contribution, modeled by the first or smaller Gaussian, as being made by the fraction of the photon beam that is properly focused in the intended position by the KB mirrors. Fig. 6 ▸ summarizes the beam size results obtained from the WFS and CSD characterization, where ‘beam size’ is the FWHM of the narrow contribution, or first Gaussian, compared with the beam size evaluated from the ray-tracing simulations performed assuming a source with no longitudinal extension [see Figs. 3 ▸(*a*) and 4 ▸(*a*)]. The results from both characterizations reproduce very well on a quantitative level the results from the simulations. The CSD characterization was moreover performed over a relatively broad photon energy range, reproducing experimentally the decreasing trend of the simulated beam size as a function of photon energy, which is mainly due to the decreasing relevance of diffraction effects.

Our experimental results show that even with state-of-the-art high-quality focusing mirrors, the beam intensity profile in the focal position can be different from an ideal micrometre-sized Gaussian. Based on ray-tracing simulations, we can assess that the contribution from aberrations due to the mirror surface quality is marginal, and the shape is likely due to the non-ideal properties of the beam source, which becomes significantly extended in the longitudinal direction when the FEL saturation conditions are reached before the last undulator cell.

This feature, which should be considered to be common to most of the XFEL experiments performed, optimizing the photon yield from the undulator, has a relevant impact on non-linear studies, which require high photon density conditions. The presence of a broad low-intensity pedestal in the beam spatial distribution, modeled as a double Gaussian spatial profile (Toyota *et al.*, 2019 ▸), has already been observed in gas-phase atomic and molecular experiments, with good agreement between experiment and theory (Toyota *et al.*, 2019 ▸; Murphy *et al.*, 2014 ▸; Rudenko *et al.*, 2017 ▸; Rudek *et al.*, 2018 ▸).

## 5. Conclusions and outlook

The SQS instrument at the European XFEL started user operation at the end of 2018, after a few weeks of commissioning dedicated to the basic equipment, with subsequent effort aimed at expanding the offer in equipment and methodologies. The beam transport system has been designed to cope with the requirement for tightly focused X-ray beams to be delivered to the instrument over a very broad range of available photon energies. The characterization for both the beamline transmission and the focusing performances, reported here, has clearly shown that these requirements have been fully met. The beam transport system has been recently upgraded with a pair of mechanically bendable KB mirrors replacing the fixed-radii substrates described in this work. The new system will allow much more flexible operation conditions, enabling, in addition, movement of the focal position of the beam to different positions along the instrument, so allowing the simultaneous installation of different end-stations.

The experimental data shown here are available at https://doi.org/10.22003/XFEL.EU-DATA-002310-00.

## Figures and Tables

**Table 1:** FEL beam parameters The photon energy ranges are limited for each electron energy set point on the low end by the minimum size of the undulator gap, on the high end by the lack of yield due to the open gap. As such, the lower limit is a hard one, whereas the higher one depends on the self-amplified spontaneous emission level. The source size and divergence FWHM are calculated according to the expressions size = 6log(6000λ [nm]) µm and divergence = 14.1/*h*ν [keV]^0.75^ µrad, respectively (Sinn *et al.*, 2011 ▸).

Electron beam energy (GeV)	16.5	14	11.5	8
Photon energy (keV)	0.92–4.0	0.66–2.9	0.5–1.96	0.26–0.95
Source size, FWHM (µm)	57 µm @ 0.5 keV			
	51 µm @ 1.5 keV			
	48 µm @ 2.5 keV			
Source divergence, FWHM (µrad)	24 µrad @ 0.5 keV			
	10 µrad @ 1.5 keV			
	7 µrad @ 2.5 keV			
Pulses s^−1^	10000 (achieved), 27000 (design)			
Pulse energy (mJ)	12 (achieved), 10 (design)			

**Table 2:** Parameters describing the beamline mirrors Beam footprints, given as FWHM, on the mirrors are not necessarily round; the given values are to be intended for the respective orientation (*e.g.* for HFM the beam given footprint is horizontal). Slope error values [root mean square (RMS)] for the tunnel mirrors are from Gerasimova *et al.* (2022 ▸).

	Distance from source (m)	Clear aperture (w × l) (mm)	Incidence angle (mrad)	Shape	Orientation	Curvature radius (km)	Tangential slope error RMS (nrad)	Beam footprint at *h*ν = 0.5, 1.6, 2.7 keV (mm)
M1	281	20 × 800	9, 13, 20	Planar	H	–	56	6.5, 2.7, 1.9
M2	283.9	20 × 800	9, 13, 20	Spherical (bendable)	H	15.03, 10.40, 6.76	58	6.5, 2.7, 1.9
M3-LEPM	300	20 × 500	20	Spherical (fixed)	V	7.62	63	6.8, –, –
M3-HEPM	300	20 × 500	9	Spherical (fixed)	V	16.49	37	–, 2.9, 2.0
M4	301	20 × 500	9, 20	Planar	V	–	140	6.8, 2.9, 2.0
HFM	444.8	20 × 600	9.15	Elliptical (fixed)	H	0.69	81	5.1, 2.2, 1.5
VFM	446.1	20 × 400	9.24	Elliptical (fixed)	V	0.415	71	3.1, 1.3, 0.9

**Table 3:** Best-fitting parameters modeling the experimental CSDs measured at photon energies between 1200 and 1700 eV with the double Gaussian superposition described in the text

*h*ν (eV)	*w* (µm)	*f*_*r*_	*w*_*r*_
1200	1.55	0.65	2.2
1250	1.70	0.60	2.0
1300	1.70	0.55	2.0
1350	1.45	0.70	2.2
1400	1.48	0.60	2.2
1450	1.43	0.65	2.2
1500	1.41	0.65	2.2
1550	1.40	0.65	2.2
1600	1.26	0.70	2.4
1650	1.26	0.70	2.4
1700	1.25	0.75	2.4
